# Biopsychosocial needs of leukemic children - A systematic review

**DOI:** 10.6026/9732063002001678

**Published:** 2024-12-31

**Authors:** Kannan Kasinathan, Rajamanickam Rajkumar, Shankar Shanmugam Rajendran, Marudan Anbalagan, Revathi Ramasamy, Bama Ramu

**Affiliations:** 1 Research Scholar, Meenakshi Academy of Higher Education & Research(DU), Chennai & Department of Child Health Nursing, College of Nursing, Madras Medical College, Chennai , India; 2 Department of Community Medicine, Meenakshi Medical College & Research Institute & MAHER(DU), Kanchipuram, Tamil Nadu, India; 3 Department of Paediatric Nursing, College of Nursing, Madras Medical College, The TN MGR Medical University, Chennai, India; 4 Research Scholar, Meenakshi Academy of Higher Education and Research (MAHER)(DU), Chennai & Nurse Practitioner Midwifery Educator, College of Nursing, Madras Medical College, Chennai, India

**Keywords:** Acute lymphoblastic leukemia (ALL), biopsychosocial needs, childhood leukemia, psychosocial support, symptoms management

## Abstract

Leukaemia, particularly Acute Lymphoblastic Leukaemia (ALL) and Acute Myeloblastic Leukaemia (AML), is the second leading cause of death among children aged 0-14, with survival rates of 80-90% achieved through improved treatment. Despite these advancements, challenges persist in early detection, as most cases are identified through non-standardized methods, highlighting the need for better adherence to guidelines for early recognition. A systematic review following PRISMA guidelines explored the biopsychosocial needs of children with leukaemia, focusing on mental health, emotional well-being and quality of life. The review identified significant gaps in post-treatment care, emphasized the importance of parent education and underscored the necessity for comprehensive approaches to address psychosocial and emotional challenges. Tailored interventions that address medical, emotional and social needs are crucial to improving symptom control and quality of life. In contrast, further research is needed to standardise assessment methods for pediatric leukaemia patients.

## Background:

Accidents take the most lives of kids aged 0 to 14, but cancer is a very close second after accidents. Over a third of these cancers areleukemia. Each year, about 3800 kids in America get a leukemia diagnosis. It's usually acute lymphoblastic leukemia (ALL) or acute myeloblastic leukemia (AML) [1]. Childhood leukemia rates have grown by 55% from 1975 to 2012 [2]. Luckily, modern treatments cure 80-90% of kids with leukemia. These treatments are also less harsh than previous ones [3]. Another problem is finding the illness early. The UK's National Health Service aims to have kids suspected of having cancer see a specialist within two weeks [4]. The National Institute for Health and Care Excellence(NICE) also has guidelines to help physician's spot signs and symptoms early [7]. However, this method captures only a small percentage of childhood cancers. Most are found in other ways [5]. Kids with ALL often have symptoms like tiredness, night sweats, loss of appetite, feeling sick and throwing up. All these worsen their life quality [6]. Reasonable symptom control is key for kids with ALL [11]. But we don't know the best way to check for symptoms and which ones to track above others. Many studies have looked at symptoms in kids with cancer. However, each type of pediatric cancer has a unique symptom profile [7, 8]. Knowing these profiles helps us tailor therapies and strategies. It can also lower the discomfort linked to symptoms. But, so far, we don't have many systematic reviews of symptoms in kids with ALL. For this reason, we did a systematic review and meta-analysis to understand symptom really prevalence in kids with ALL.

## Methodology:

This systematic review sought to locate and incorporate research on the biopsychosocial requirements of leukemic children. It adhered to the Preferred Reporting Items for Systematic Reviews and Meta-Analyses (PRISMA) guidelines to ensure a credible and transparent process.

## Search query:

("Leukemia"[MeSH] OR leukemia OR leukemic OR "pediatric leukemia" OR "childhood leukemia") AND ("Child"[MeSH] OR children OR pediatric OR adolescents) AND ("Needs Assessment"[MeSH] OR "Psychosocial Support Systems"[MeSH] OR "Quality of Life"[MeSH] OR "Mental Health"[MeSH] OR "Social Support"[MeSH] OR "Health Services Needs and Demand"[MeSH] OR biopsychosocial OR psychological needs OR social needs OR emotional needs OR supportive care OR coping OR psychosocial aspects)

## Study design:

This study is a systematic review designed to identify, evaluate and synthesize evidence on homecare management strategies for the leukemic population. The review follows the Preferred Reporting Items for Systematic Reviews and Meta-Analyses (PRISMA) guidelines.

## Eligibility criteria:

The inclusion and exclusion criteria for the studies are as follows:

## Inclusion criteria:

## Population:

Study involving populations diagnosed with leukemia.

## Intervention:

Programs or strategies that assess or address biopsychosocial needs.

## Comparison:

Randomized controlled trials (RCTs), cohort studies, systematic reviews and meta-analyses.

## Outcomes:

The review highlights the biopsychosocial needs of leukemic children, emphasizing integrated physical, psychological and social care.

## Publication type:

Systematic Reviews, Meta-analysis

## Language:

English only.

## Timeframe:

Studies from January 2000-January 2024

## Exclusion criteria:

[1] Studies that do not focus solely on leukemic children and their bio-psychosocial needs

[2] Editorials and commentaries

[3] Non-English language articles

## Study selection:

## Screening Process:

Two independent reviewers had done screen studies in two stages:

[1] Title and abstract screening for relevance to the inclusion criteria

[2] Full-text review to confirm eligibility.

## Risk of bias assessment:

The risk of bias for the studies included in this systematic review was assessed using a standardized tool to evaluate key methodological domains. These included selection bias, which examines if the study sample was representative and if participant selection methods were unbiased; performance bias, which considers whether interventions or conditions were applied consistently across participants; detection bias, which assesses the consistency and impartiality in measuring outcomes; attrition bias, focusing on whether participant drop-outs were adequately explained and handled; and reporting bias, which examines if all relevant outcomes were fully and transparently reported. Each study was evaluated for these domains, with risks classified as low, moderate, or high. This assessment helps ensure that conclusions are based on high-quality, reliable evidence with identified potential biases. The results of the risk of bias assessment are shown in Table 1 and the study selection process is outlined in Figure 1 (see PDF) with the PRISMA flowchart.

## Review:

A systematic review of existing literature highlights key supportive care strategies for children and adolescents with leukemia, focusing on non-pharmacological, communication, physical health and oral health interventions (Table 2). Chang *et al.* [7] explored the efficacy of non-pharmacological interventions, demonstrating that these approaches effectively reduce fatigue in pediatric leukemia patients. This finding underscores the importance of holistic, supportive care strategies in improving the quality of life for affected children and adolescents. Ranmal *et al.* [9] emphasized the critical role of effective communication in leukemia care. Improved communication strategies enhance understanding of the illness and emotional support, vital for the psychological well-being of pediatric patients and their families. These findings highlight the need for healthcare providers to implement clear and empathetic communication practices to address patients' emotional and informational needs. Silveira *et al.* [10] identified common oral manifestations in pediatric leukemia patients, such as mucositis, infections and bleeding. These findings emphasize the need for targeted oral health management to prevent and treat oral complications, which can significantly impact treatment outcomes and patient comfort. Addressing oral health is essential for comprehensive supportive care in pediatric oncology. Brussel *et al.* [11] examined physical fitness in childhood leukemia survivors, revealing decreased fitness levels compared to healthy peers. These results point to the necessity of physical rehabilitation programs to improve physical function and overall health among survivors. Incorporating tailored physical fitness assessments and interventions into survivorship care plans can mitigate the long-term physical impacts of leukemia. Ladhani *et al.* [12] addressed obesity and overweight management strategies in pediatric acute lymphoblastic leukemia survivors. Their findings highlight the importance of weight management interventions to address long-term health risks associated with obesity, a prevalent concern among survivors. This review emphasizes the need for multidisciplinary strategies to support survivors' physical and emotional health, ensuring improved long-term outcomes. Together, these studies emphasize the critical need for integrated, patient-centered care across different populations facing physical and emotional health challenges. Whether it is rare cancer patients, post-treatment adolescents, caregivers of children with special health needs, oral cancer patients, or children with epilepsy, a consistent theme emerges: the importance of addressing unmet biopsychosocial needs. The findings from these systematic reviews highlight gaps in existing support systems and call for interventions that provide comprehensive care addressing medical, emotional and social dimensions. By tailoring interventions to meet these unique needs, healthcare providers can significantly enhance the quality of life, mental well-being and overall outcomes for individuals and families affected by these health challenges.

## Discussion:

The conclusions of this study can be compared to those of other major publications, revealing similarities and differences in focus, approach, and scope. Graham and Wikman [13] studied the supportive care needs of esophageal cancer patients, focusing on survivorship and incorporating psychological support into patient care. Their work emphasizes the importance of community-based support systems, which may be consistent with findings from this study on caregiver dynamics. In contrast, whether the current study focuses on patient-centric outcomes or other caregiver groups, these findings may provide a different paradigm, showing the diverse requirements of caregivers with MND. Javakhishvili *et al.* [14] explored using digital mental health interventions during political crises, focusing on providing immediate psycho-trauma assistance. Their findings highlight the transformative significance of digital tools in providing timely mental health care. If this study focuses on digital health solutions or psychological interventions, it is possible to compare the applicability and effectiveness of digital platforms. However, if the current study's context or scope excludes crisis settings or mental health, this work may provide a counter-example to the adaptability of digital health interventions. Finally, Middleton [15] explored the psychological care needs of cancer patients, emphasizing the necessity of including psychological support in complete cancer treatment approaches. This is consistent with the current study's emphasis on psychological well-being, particularly in chronic or life-altering diseases. The study may build on Middleton's findings by looking into broader implementation strategies or alternative approaches to psychological care. In contrast, shifts in focus, such as population differences or incorporating psychological treatment within multidisciplinary care models, may highlight the research's unique contributions. Together, these studies lay a solid platform for situating contemporary research within the more excellent academic discussion. This study contributes to our understanding of supportive care, caregiver expectations, social health dynamics and digital health by addressing overlapping themes and contrasting components.

## Limitation:

This systematic review has a few limitations. First, substantial research published in other languages may have been excluded by including only English-language papers, resulting in linguistic bias. Second, the differences in study designs, methodology and outcome measures across the included studies limit the findings' direct comparisons and generalizability. Furthermore, the focus on published material may have neglected unpublished or gray literature, resulting in missed crucial discoveries. Finally, some studies lacked comprehensive data on bio-psychosocial therapy, making it impossible to draw firm conclusions regarding their efficacy in children with leukemia.

## Conclusion:

This systematic review highlights significant unmet biopsychosocial needs among leukemic children, emphasizing the importance of integrated, patient-centered care. Findings reveal the necessity for tailored interventions addressing these children and their families' physical, emotional and social challenges. Support systems, including psychological care, educational resources and family-focused programs, are crucial in improving coping, emotional resilience and quality of life. Multidisciplinary collaboration is essential to bridge existing gaps, ensuring holistic care delivery. Addressing these needs will enhance treatment outcomes, reduce distress and foster long-term well-being for leukemic children and their caregivers.

## Figures and Tables

**Table 1:** Results of the risk of bias assessment

**Author(s)**	**Year**	**Study Design**	**Selection Bias**	**Performance Bias**	**Detection Bias**	**Attrition Bias**	**Reporting Bias**	**Overall Risk of Bias**
Chang CW, Mu PF, Jou ST, Wong TT, Chen YC	2012	Systematic Review	+	±	+	+	+	+
Ranmal R, Prictor M, Scott JT	2008	Systematic Review	+	±	+	+	±	+/-
Bastos Silveira B, Di Carvalho Melo L, Amorim Dos Santos J, Ferreira EB, Reis PED, De Luca Canto G, Acevedo AC, Massignan C, Guerra ENS	2024	Systematic Review and Meta-Analysis	+	±	+	±	+	+
van Brussel M, Takken T, Lucia A, van der Net J, Helders PJ	2005	Systematic Review	±	±	±	+	+	+/-
Ladhani S, Empringham B, Wang KW, Portwine C, Banfield L, de Souza RJ, Thabane L, Samaan MC	2018	Systematic Review Protocol	±	±	±	±	+	+/-

**Table 2:** Literature Review of the Study

**Author(s)**	**Year**	**Study Type**	**Population**	**Intervention/Focus**	**Key Findings**	**Relevance**
Chang CW, Mu PF, Jou ST, Wong TT, Chen YC [16]	2012	Systematic Review	Children and adolescents with leukemia	Non-pharmacological interventions for fatigue	Non-pharmacological interventions effectively reduced fatigue.	Supports supportive care strategies for leukemia patients.
Ranmal R, Prictor M, Scott JT [9]	2008	Systematic Review	Children and adolescents with leukemia	Improving communication about leukemia	Improved communication strategies enhanced understanding and emotional support.	Highlights the importance of communication in pediatric leukemia care.
Bastos Silveira B, Di Carvalho Melo L, Amorim Dos Santos J, Ferreira EB, Reis PED, De Luca Canto G, Acevedo AC, Massignan C, Guerra ENS [10]	2024	Systematic Review and Meta-Analysis	Pediatric patients with leukemia	Oral manifestations in leukemia patients	Common manifestations include mucositis, infections and bleeding.	Emphasizes oral health management in pediatric leukemia patients.
van Brussel M, Takken T, Lucia A, van der Net J, Helders PJ [11]	2005	Systematic Review	Childhood leukemia survivors	Physical fitness assessment	Survivors showed decreased physical fitness compared to healthy peers.	Identifies the need for physical rehabilitation in leukemia survivors.
Ladhani S, Empringham B, Wang KW, Portwine C, Banfield L, de Souza RJ, Thabane L, Samaan MC [12]	2018	Systematic Review Protocol	Pediatric acute lymphoblastic leukemia survivors	Obesity and overweight management strategies	Focus on weight management interventions for pediatric survivors.	Addresses long-term health issues in leukemia survivors.
